# Real-Time Packing Behavior of Core-Shell Silica@Poly(*N*-isopropylacrylamide) Microspheres as Photonic Crystals for Visualizing in Thermal Sensing

**DOI:** 10.3390/polym8120428

**Published:** 2016-12-10

**Authors:** Karthikeyan Manivannan, Yi-Shen Huang, Bohr-Ran Huang, Chih-Feng Huang, Jem-Kun Chen

**Affiliations:** 1 Department of Materials Science and Engineering, National Taiwan University of Science and Technology, 43, Sec 4, Keelung Road, Taipei 106, Taiwan; kmanivannan2012@gmail.com; 2 Department of Chemical Engineering, National Chung Hsing University, 250 Kuo Kuang Road, Taichung 402, Taiwan; yishen617@gmail.com; 3 Graduate Institute of Electro-Optical Engineering and Department of Electronic Engineering, National Taiwan University of Science and Technology, 43, Sec 4, Keelung Road, Taipei 106, Taiwan; huangbr@mail.ntust.edu.tw

**Keywords:** core-shell, SiO_2_ microsphere, structural color, photonic crystals, polymerization

## Abstract

We grafted thermo-responsive poly(*N*-isopropylacrylamide) (PNIPAM) brushes from monodisperse SiO_2_ microspheres through surface-initiated atom transfer radical polymerization (SI ATRP) to generate core-shell structured SiO_2_@PNIPAM microspheres (SPMs). Regular-sized SPMs dispersed in aqueous solution and packed as photonic crystals (PCs) in dry state. Because of the microscale of the SPMs, the packing behavior of the PCs in water can be observed by optical microscopy. By increasing the temperature above the lower critical solution temperature (LCST) of PNIPAM, the reversible swelling and shrinking of the PNIPAM shell resulted in dispersion and precipitation (three-dimensional aggregation) of the SPM in aqueous solution. The SPMs were microdispersed in a water layer to accommodate the aggregation along two dimensions. In the microdispersion, the SPMs are packed as PCs with microscale spacing between SPMs below the LCST. When the temperature is increased above the LCST, the microdispersed PCs exhibited a close-packed arrangement along two dimensions with decreased spacing between SPMs. The change in spacing with increasing temperature above the LCST resulted in a color change from red to blue, which could be observed by the naked eye at an incident angle. Thus, the SPM array could be applied as a visual temperature sensor.

## 1. Introduction

Core-shell composite materials consist of a shell structure covering a core structural area and are typically spherical in shape. They may be composed of a variety of materials including polymers and inorganic solids such as silica, gold, and silver [1]. In the past year, core-shell nanostructured polymers have attracted much attention because of their various potential applications in catalysis [2], display technologies [3,4], actuation systems [5,6], biosensors [7,8], and electronics [9,10].

Photonic crystals (PCs), which are composed of periodic arrays of contrasting refractive index materials, have found an increasing number of applications in ever-widening areas of science and technology such as gas-based nonlinear optics [11,12]. Recently, many techniques have been developed to prepare thermoresponsive PCs using poly(*N*-isopropylacrylamide) (PNIPAM) particles for almost a decade ago [13]. Various stimuli-responsive polymers have the ability to switch the optical properties of core-shell silica microspheres, such as PCs including colloidal crystals [14,15]. The optical properties of these PCs can be switched by external triggers, such as change of temperature, light, solvent, ionic strength, magnetic fields, mechanical stress, electric fields [16,17], and nanostructure arrays [18]. Moreover, temperature-tunable PCs with hierarchical structures can be fabricated by combining natural PCs with thermoresponsive polymers. Hence, thermoresponsive PNIPAM-functionalized silica particles, obtained by surface modification processes, have been applied in many fields including biomedical, drug delivery, biosensing, and tissue imaging systems [19,20,21,22,23,24].

**Figure 1:**
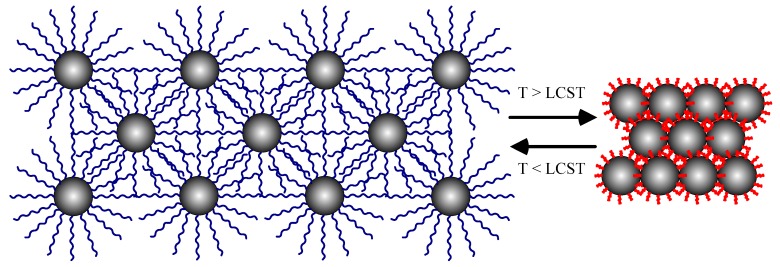
Cartoon representation of the SPMs array in aqueous layer, displaying a microdispersed state and 2D aggregated state below and above the LCST, respectively. Blue and red chains represent the extended and collapsed PNIPAM, respectively.

Smart-polymers based on PNIPAM can be effective temperature-triggered flocculants in the minerals industry [25,26]. PNIPAM undergoes phase separation in aqueous solutions above its lower critical solution temperature (LCST) of 32 °C. In suspensions of silica particles coated with the polymer, this phase change causes attractive interactions, aggregation, and rapid settling. Below the LCST, the polymer acts as a dispersant, because of repulsive particle interactions [27,28]. In the preparation of opal photonic crystal films, two types of thermoresponsive polymers can be used as forming materials: poly(diethylene glycol methyl ether methacrylate) and PNIPAM [29]. When scattering mode is used to detect the photoluminescence, the emission comes from the top surface of the colloidal crystals and is collected by the detector; thus, the stop band of the resulting colloidal crystal exerts a minor influence on the resulting spectra [30]. If the monodisperse spherical particles are spread quickly onto a surface, the colloidal particles self-assemble into randomly oriented, hexagonally arranged microcrystallites [31,32]. The applications of SiO_2_@polymer and hydrogel nanoparticles in the fabrication of photonic crystals have been widely studied [33]. However, the nanoparticles could be observed only under dry conditions, such as in the vacuum environment used for scanning electron microscope (SEM) and transmission electron microscope (TEM) measurements. The real-time packing behavior of particles has been rarely reported. In this study, thermoresponsive PNIPAM brushes were grafted onto silica microspheres to generate core-shell structured SiO_2_@PNIPAM microspheres (SPMs). Regular-sized SPMs dispersed in aqueous solution and packed as PCs after water removal. Because of the micro scale of the SPMs, the real-time packing behavior of the PCs during water removal could be observed by optical microscopy (OM). The PNIPAM shell facilitates the dispersion of the SPMs resulting in an increased spacing between SPMs on the surface (Figure 1). The spacing between SPMs could be tuned by adjusting the temperature to change the optical properties of the PCs. The reversible swelling and shrinking behaviors of the SPM array related to the surface optical properties of the PCs were investigated during the temperature changes.

## 2. Experimental Section

### 2.1. Materials

Silica microspheres (size: ca. 1 μm) were purchased from Polysciences (Warminster, PA, USA); *N*-isopropylacrylamide, (NIPAM, 99%), was purified by recrystallization from the *n*-hexane, CuBr (98%), CuBr_2_ (99%), and aminopropyltriethoxysilane (APTES), 1,1,4,7,7-pentamethyldiethylenetriamine (PMDETA, 99%), 2-bromoisobutyryl bromide (BiBB, 97%), were purchased from Acros Organics (Geel, Belgium). All the other reagents were purchased from Sigma-Aldrich (St. Louis, MO, USA). All the solvents used in this study are reagent grade and used without further purification.

### 2.2. Synthesis of PNIPAM-Functionalized Silica Microparticle

**Scheme 1:**
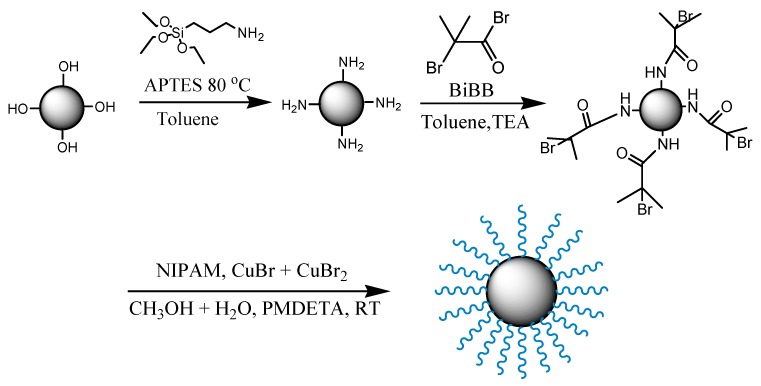
Schematic representation of the synthesis of hybrid silica microspheres, grafted with thermoresponsive PNIPAM via SI ATRP. The blue chains indicate the tethered PNIPAM.

Scheme 1 shows the preparation of PNIPAM grafted from silica microspheres by ATRP initiator-functionalized silica microspheres. Briefly, the silica dispersion (800 mg SiO_2_) were placed in into a three-neck (100 mL) round-bottom flask which contains dry toluene (20 mL) and equipped with a magnetic stir bar, also fitted with a reflux condenser. After addition of APTES (1 mL), the reaction mixture was refluxed for 20 h at 80 °C under N_2_ atmosphere. After completion of reaction, the reaction mixture were cooled to room temperature, then the particles were isolated by centrifugation at 6000 rpm for 15 min. After that, the supernatant was redispersed into the toluene and centrifuge several times using dichloromethane and acetone, separately. The amine-modified silica was dried in vacuum oven at 50 °C for overnight [29]. The amino-modified silica microspheres in the toluene suspension (700 mg SiO_2_–NH_2_) was mixed with triethylamine (1.2 mL) into a 100 mL round-bottom flask. After that, the flask was cooled to 0 °C then 1 mL of BiBB was added dropwise for 1 h and elevated to room temperature for an additional 22 h. The microspheres were purified and isolated following similar procedures for the synthesis of amino-functionalized silica microspheres. Initiator-functionalized silica microparticle (100 mg), PMDETA (0.124 mL), CuBr (15 mg), NIPAM (1 g), and MeOH/DI water (1:1 vol.) were added to a round-bottom flask equipped with a magnetic stir bar. The silica microspheres mixture was degassed by three freeze-pump-thaw cycles to graft NIPAM from silica microsphere for 12 and 16 h [34]. Next, the microspheres were isolated and purified following the procedure mentioned above, by centrifugation with ethanol and methanol. The as-prepared SPMs were incubated in water below and above the LCST for 30 min, and then lyophilized to analyze the thickness and surface chemical composition.

### 2.3. Characterization

The SPMs were analyzed by using Fourier transformer infrared spectroscopy (FT-IR: PerkinElmer lambda 25 spectrophotometers, Digilab-FTS1000, (Waltham, MA, USA). Thermogravimetric analysis (TGA) was performed on a Q500 TA instrument (New Castle, DE, USA) at a heating rate of 10 °C/min from room temperature to 800 °C under nitrogen atmosphere. The morphology of the samples was characterized by using a field-emission scanning electron microscope (SEM: JSM-6500F, JEOL, Peabody, MA, USA) and field-emission transmission electron microscope (TEM: Philips Tecnai G2 F20, Hillsboro, OR, USA). Top-view images of the samples were obtained by optical microscope (OM: Nikon Eclipse FN1, DPSS 532 nm Laser, Melville, NY, USA). 

## 3. Results and Discussion

**Figure 2:**
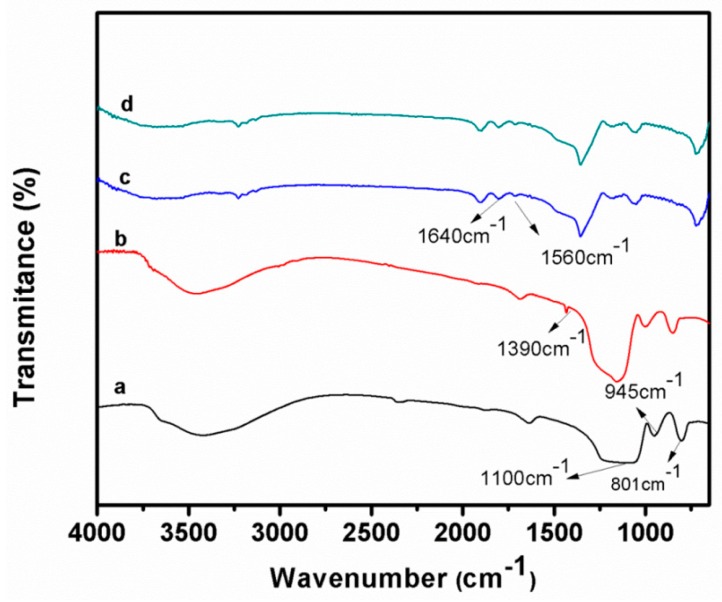
FT-IR spectra of (**a**) bare silica microspheres; (**b**) BiBB-functionalized silica microspheres; and SPMs (**c**) below and (**d**) above the LCST.

The FT-IR spectra of bare and BiBB-functionalized silica microspheres are clearly different (Figure 2). In the spectrum of the bare silica microspheres (Figure 2a), the absorption peaks’ characteristic of tetrahedron silica structures were observed at 1100 cm^−1^ (Si–O stretching) and 465 cm^−1^ (Si–O bending); the peaks related to the Si–OH bending at 945 cm^−1^ and Si–O–Si bending at 801 cm^−1^ were also detected [35]. After amidation, the C–H stretching and bending vibration bands at 1390 and 2950 cm^−1^, respectively, appeared (Figure 2b) [36]. Moreover, in the spectrum of the hybrid silica microparticles embedded with PNIPAM chains, attained by surface-initiated ATRP for 16 h, amide bands at 1640 and 1560 cm^−1^ were observed, which could be attributed to the C=O stretching and N–H stretching, respectively, of the SPMs below the LCST (Figure 2c) [37]. In addition, in the spectrum of the SPMs above the LCST, the characteristic PNIPAM peak did not change significantly, indicating the stability of the PNIPAM grafts. These observations confirmed the successful PNIPAM grafting from the initiator-modified particles.

To support the characterization of the functionalized silica microspheres, alternatively, the surfaces were examined by X-ray photoelectron spectroscopy (XPS, Waltham, MA, USA) (see the Figure S1 in the Supplementary Materials). For bare SiO_2_ (Figure S1a), the characteristic signals of silicon (at 103 eV (Si2p) and 155 eV (Si2s)) and oxygen (at 532 eV (O1s)) were clearly detected. In the spectrum of SiO_2_-BiBB (Figure S1b), the characteristic peaks of nitrogen (at 400 eV (N1s)) and bromine (at 68 eV (Br3d)) appeared. For SiO_2_-PNIPAM (Figure S1c), the characteristic nitrogen peak appeared, and the silicon and bromine peaks disappeared, as expected. These results confirm the successful modification of SiO_2_ microspheres to form core-shell structures of SiO_2_@PNIPAM.

**Figure 3:**
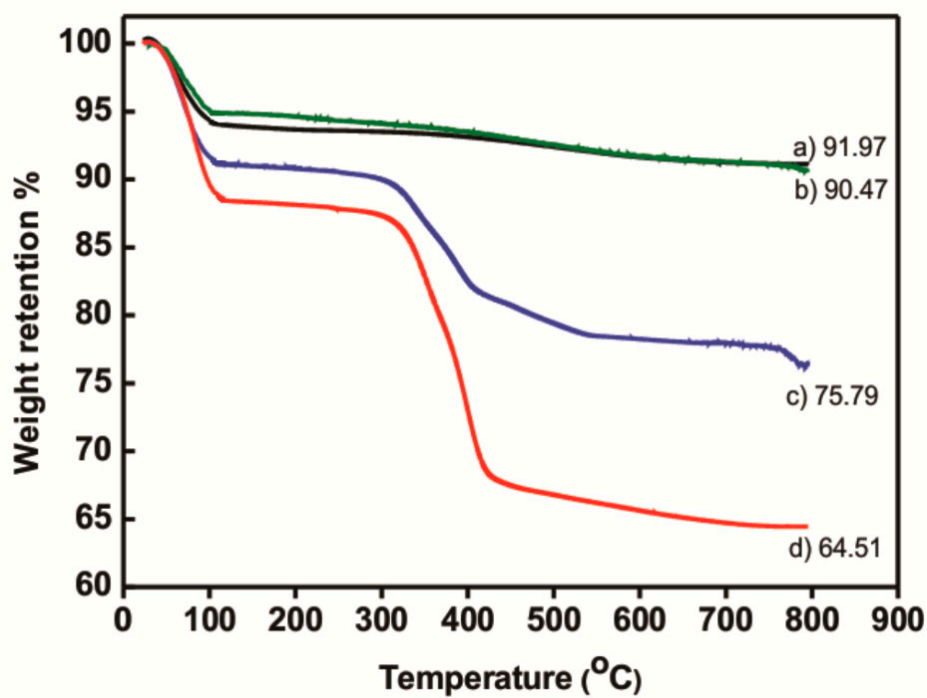
TGA of (**a**) bare silica microspheres; (**b**) BiBB-functionalized silica microspheres and silica microspheres possessing grafted PNIPAM brushes through SI ATRP for (**c**) 12 and (**d**) 16 h.

TGA analysis of silica microspheres, SiO_2_-Br, and SPMs (polymerization time: 12 and 16 h), shown in Figure 3, reveals a weight loss for all samples below 100 °C due to desorption of water molecules. Bare silica microspheres were analyzed (Figure 3a) for comparison, to confirm the attachment of BiBB at 800 °C, a weight retention difference of ~1.5% between amino- and BiBB-functionalized silica microspheres (Figure 3b) was observed, and the monomer conversion (wt %) to PNIPAM-grafted silica microspheres with a polymerization time of 12 and 16 h was ~16.8% and ~27.46%, respectively (Figure 3c,d) [35].

**Figure 4:**
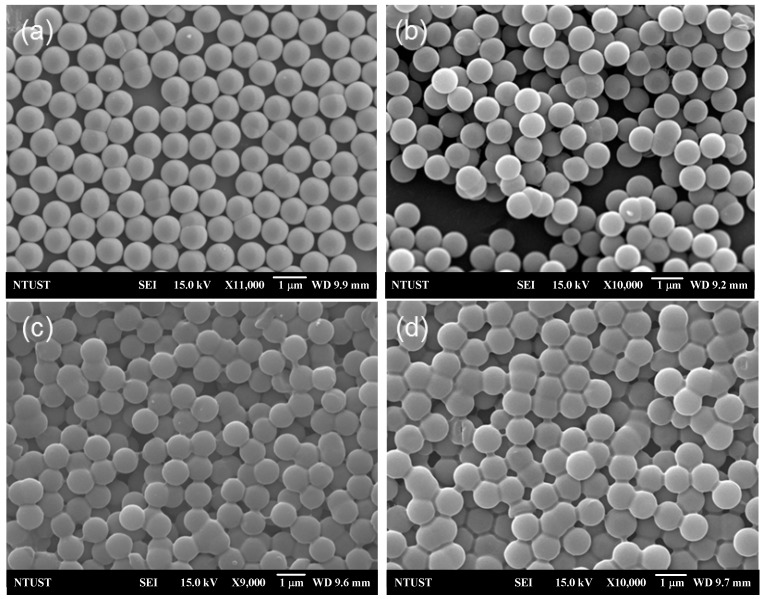
SEM images of (**a**) bare silica microspheres; (**b**) BiBB-functionalized silica microspheres; and SPMs incubated (**c**) below and (**d**) above the LCST.

SEM images were recorded for silica microspheres, SiO_2_-Br, and SPMs, below and above the LCST (Figure 4). The average diameter of the initiator-functionalized silica microspheres remained unchanged at 1 μm. After PNIPAM grafting from the surface through SI ATRP, below and above the LCST, microspheres showed a neck structure, as illustrated in Figure 4c,d. A slight increase in microsphere size could be detected; however, the swelling and shrinking behavior of SPMs in the dry state could not be clearly observed by SEM.

**Figure 5:**
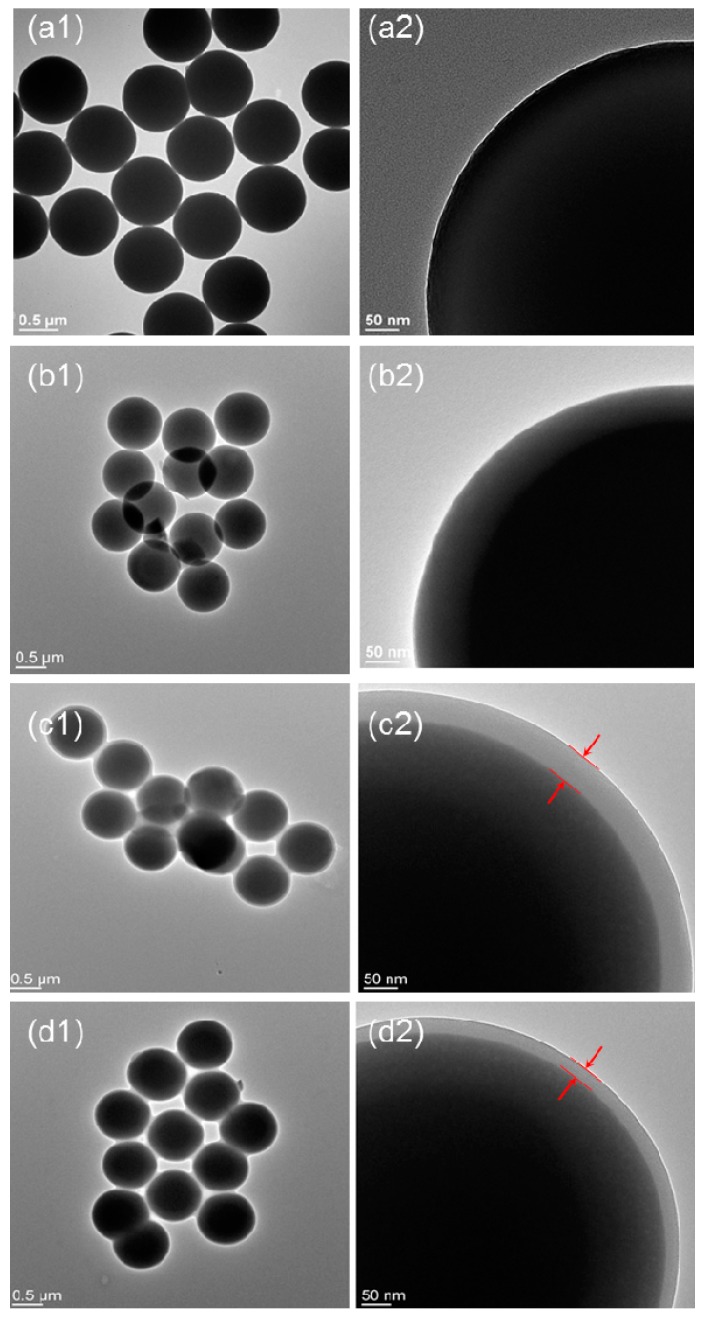
TEM images of (**a**) bare silica microspheres; (**b**) BiBB-functionalized silica microspheres; and SPMs incubated (**c**) below and (**d**) above the LCST. The red arrows indicate the thickness change of PNIPAAM below and above LCST, repsectively (1: large scale of samples; 2: zoom-in scale of the coressponding samples).

TEM observations confirmed the average diameter of 1 μm for silica microspheres (Figure 5a) and the slight increase in microsphere size upon PNIPAM grafting. The increase in size was attributed to the APTES modification of the amine-functionalized silica microspheres. Subsequent functionalization by amidation of amine-functionalized silica microspheres with BiBB was also observed by TEM (Figure 5b). The TEM images of the silica microspheres grafted with PNIPAM for 16 h below the LCST clearly show a polymer layer with a thickness of 50 nm covering the silica core (Figure 5c). The thickness of the PNIPAM shell decreased from 50 to 35 nm with increasing temperature above the LCST [36]. It should be noted that the SPMs were lyophilized after incubation below and above the LCST; thus, the thickness change of the PNIPAM shell may not be very precise. Nevertheless, the thickness change revealed shrinking of the PNIPAM shell of the SPMs. Hence, PNIPAM proved to be an excellent shell-forming material for silica core microspheres for the fabrication of hybrid SiO_2_@PNIPAM.

**Figure 6:**
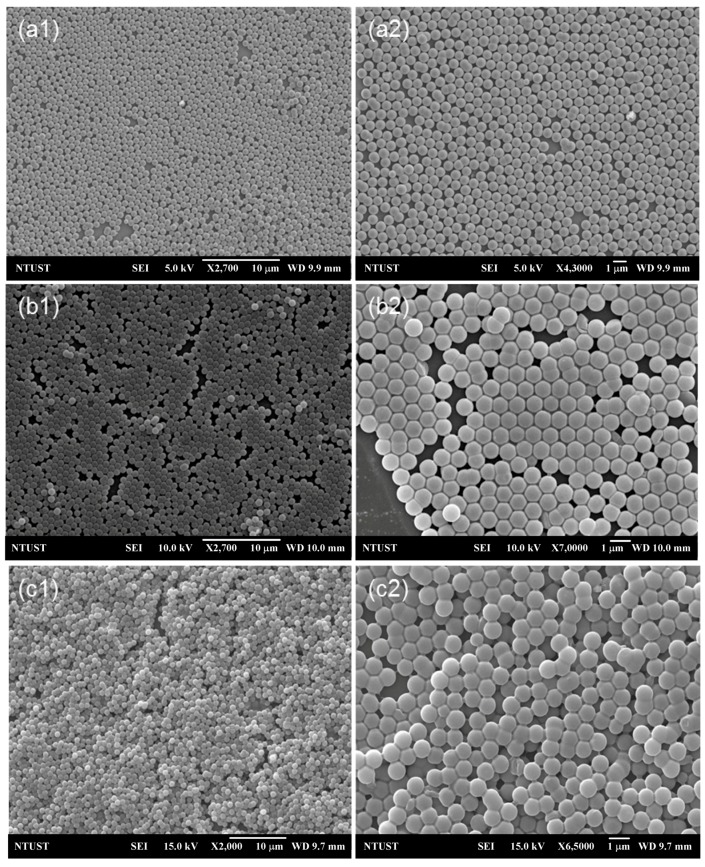
SEM images of the PCs of (**a**) bare silica microsphere array and SPMs array of (**b**) below and (**c**) above the LCST.

The prepared SPMs were used to form PC structures for OM observation. To obtain a regular 2D SPM array, the SPM solutions were dip-coated on glass slides and incubated below and above the LCST for 30 min. SEM images of the 2D SPM array were recorded below and above the LCST (Figure 6). The silica microspheres were hexagonally packed along two dimensions as PCs (Figure 6a). Below the LCST, SPMs also exhibited a close-packed arrangement and revealed superior PC properties (Figure 6b). Increasing the temperature above the LCST enhanced the adhesion between SPMs resulting in closer packing (Figure 6c). These results suggest that increasing the temperature above the LCST significantly decreased the spacing between SPMs.

**Figure 7:**
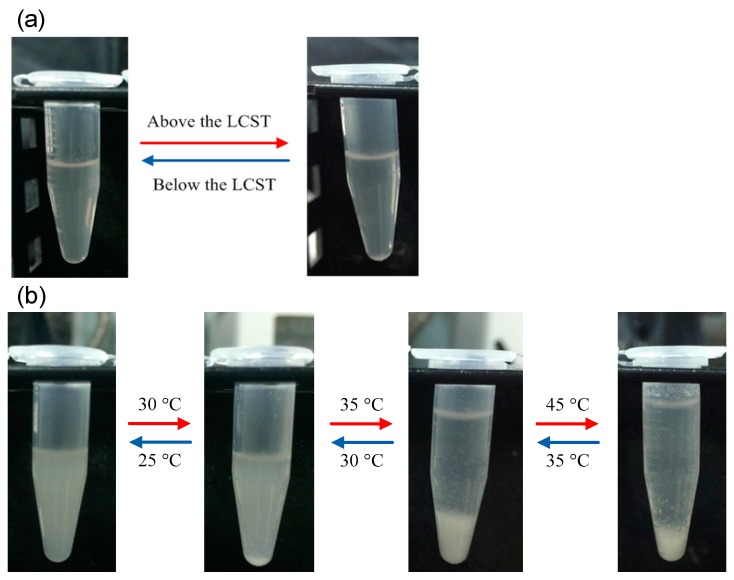
Illustrative images of (**a**) bare silica microspheres and (**b**) SPMs upon increasing the temperature above the LCST in aqueous solution.

SPMs could pack as PCs after removal of water on the surface. However, the SPM may not undergo swelling or shrinking upon temperature switch in a dry state. To observe the swelling and shrinking behavior, the SPMs must be dispersed in water. In order to observe the packing behavior in real time, water droplets of different temperatures were added dropwise into the solution, and photographs of silica microspheres and SPMs dispersing in water were taken below and above the LCST (Figure 7). The solution of bare silica microspheres exhibited a slight turbidity, which did not change upon increasing the temperature above the LCST (Figure 7a). The turbidity of the SPMs solution below the LCST significantly increased due to the PNIPAM coating, whereas above the LCST the thermoresponsive SPMs undergo a reversible phase separation resulting in aggregation: during the phase separation, the interactions between SPMs increased significantly, thus increasing the adhesion forces between SPMs. Hence, the SPMs randomly aggregated and rapidly precipitated (Figure 7b). Similarly, switching the temperature below the LCST weakened the interactions between SPMs resulting in the dispersion of SPMs. It should be noted that changing the temperature by dropwise addition of water of different temperatures resulted in volume change, as shown in Figure 7b.

**Figure 8:**
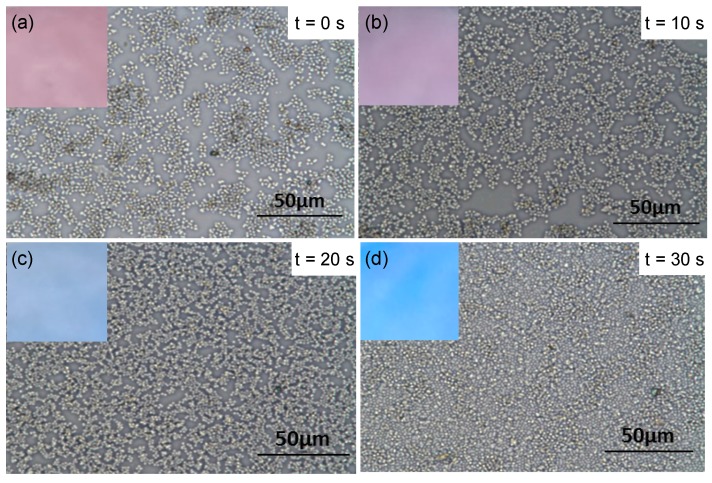
Real-time optical microscopy images of SPMs array from 0 to 30 s (i.e., images (**a**–**d**)) after increasing the temperature above the LCST.

Random aggregations of microspheres, without ordered packing, did not exhibit the properties of PCs. Thus, these microspheres were dip-coated on glass slides without water removal. To investigate the packing behavior below and above the LCST, the top-view OM images of various samples were taken. The bare silica microspheres packed densely on the surface within the water layer. As a control experiment, increasing the temperature above the LCST of PNIPAM did not significantly change the packed structure of bare silica microspheres (Figure S2, Supplementary Materials). The packing behaviors of SPMs were monitored in real-time scale in 30 s by shifting the solution temperature above the LCST (Figure 8a–d). The SPMs gradually packed along two dimensions resulting in a decrease in spacing between SPMs. The inserts in Figure 8, displaying the images of the photonic crystals of silica microspheres and SPMs, show that the bare silica microsphere array exhibited deep blue color at an observation angle of 30°. The SPM array exhibited red color below the LCST due to the larger spacing between the microspheres. When the temperature was increased above the LCST, the red color gradually turned to light blue. The diffraction peak position (*λ*_max_) of the PCs could be evaluated using the following equation:

$$k\cdot \lambda_{\text{max}}=\sqrt{\frac{3}{8}}\cdot d\cdot n_{eff}\cdot \text{sin}\;\theta$$

where *θ* is the glancing angle between the incident light and diffraction crystal planes, *n*_eff_ is the effective refractive index, and *k* is the order of the Bragg diffraction [37,38,39]. In this study, *k*, *n*_eff_, and *θ* were invariable. Thus, the only factor influencing the value of *λ*_max_ was the spacing between microspheres: an increase of spacing resulted in a red shift in the *λ*_max_. Thus, the color change from red to blue with increasing temperature above the LCST was predominantly determined by the increase in spacing. The colors of the SPM array confirmed that the microspheres could pack to form PCs in a water layer below the LCST. We define a micro-dispersion that represented an ordered dispersion with spacing in micro-scale among the SPMs in a water layer (Figure 1). The spacing between SPMs decreased along two dimensions of the ordered structures resulting in the color change. The red-to-blue color change of the SPM array with increasing temperature above the LCST could be used in visual temperature sensing [40]. Currently, we are conducting a protein-immobilized SPM for the application of H_2_O_2_ sensing based on the thermoresponsive behavior and the results will be presented systematically in a later article.

**Figure 9:**
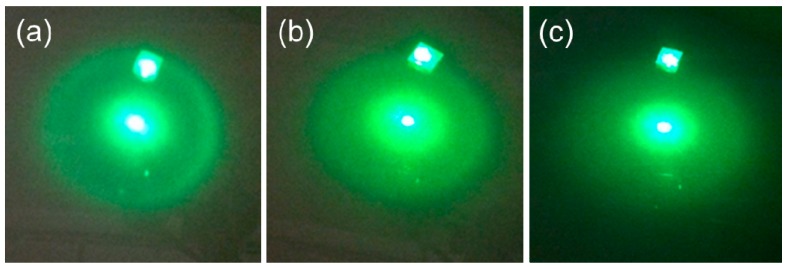
Diffraction patterns of a green laser beam traveling through the SPMs array on glass slides (**a**) below; (**b**) close to; and (**c**) above the LCST.

Moreover, the light scattering behavior of the SPM array was investigated by using a green laser beam below, close to, and above the LCST, as shown in Figure 9. The physical foundation of the instrument used is the Mie theory, which is based on Maxwell’s equations. The Mie theory is a rigorous solution for the light scattered by a spherical, homogeneous, isotropic, and non-magnetic particle in a non-absorbing medium [41]. If the particle size is much larger than the wavelength of light, the Fraunhofer diffraction theory can also give a good description of the light scattered in the near-forward directions. In the Fraunhofer diffraction theory, the scattering particle is regarded as a totally opaque disk of the same diameter, and its far-field scattering pattern is composed of a central bright spot gradually decaying from inside out and a series of concentric rings. Such scattering pattern is called the Airy disk. The Airy disk size is described by the angular radius *θ*_A_ at which the first minimum of the scattering pattern intensity occurs. It is generally accepted that the Airy disk size monotonically decreases with increasing particle size. This fine structure forms the foundation of the laser diffraction method. In this work, we focused on the change of Airy disk size with changing SPM size due to the temperature increase above the LCST. When the laser light passed through the SPM array-coated glass surface, a corona-like pattern, i.e., the Airy disk, appeared behind the glass slide. The angular radius of the Airy disk decreased with increasing temperature above the LCST, in agreement with the Fraunhofer diffraction theory. These results suggest that the Airy disk could be used to detect the dimensional change of particles in SPM arrays.

## 4. Conclusions

We synthesized a core-shell structure comprising a silica microsphere core and a PNIPAM-grafted shell. Because of their micro scale, the real-time packing behavior of these particles in solution could be observed by OM. The core-shell microspheres exhibited reversible swelling and shrinking behaviors as the temperature was increased above the LCST. The microspheres could pack in a water layer as PCs. Increasing the temperature above the LCST caused a decrease in the spacing between microspheres, which resulted in a color change of the PCs. The visual red-to-blue color change confirmed that the ordered packing of the microspheres in the water layer was retained during the temperature increase. Hence, the synthesized PNIPAM-grafted silica microspheres could provide an understanding of the packing mechanism of PCs in water, and could be used in a wide range of visual color sensor and optical applications.

## Supplementary Materials

The following are available online at www.mdpi.com/2073-4360/8/12/428/s1, Figure S1: XPS spectra of (a) bare silica particles, (b) BiBB-functioned silica particles, and (c) PNIPAM-grafted silica particles; Figure S2: Control experiment: OM images of bare silica microsphere (a) below and (b) above the LCST of PNIPAM.
