# Supplementary material for: Real-Time Packing Behavior of Core-Shell Silica@Poly(N-isopropylacrylamide) Microspheres as Photonic Crystals for Visualizing in Thermal Sensing
Source: Polymers (Basel). 2016 Dec 10;8(12):428. doi: 10.3390/polym8120428 (PMC6431998; doi:10.3390/polym8120428)
Supplement: Supplementary file 1 [file polymers-08-00428-s001.pdf]

Polymers **2016**, *8*, 428; doi:10.3390/polym8120428

Supplementary Materials: Real-time Packing Behavior of Core-Shell Silica@Poly(*N*-isopropylacrylamide) Microspheres as Photonic Crystals for Visualizing in Thermal Sensing

Karthikeyan Manivannan, Yi-Shen Huang, Bohr-Ran Huang, Chih-Feng Huang *, and Jem-Kun Chen *

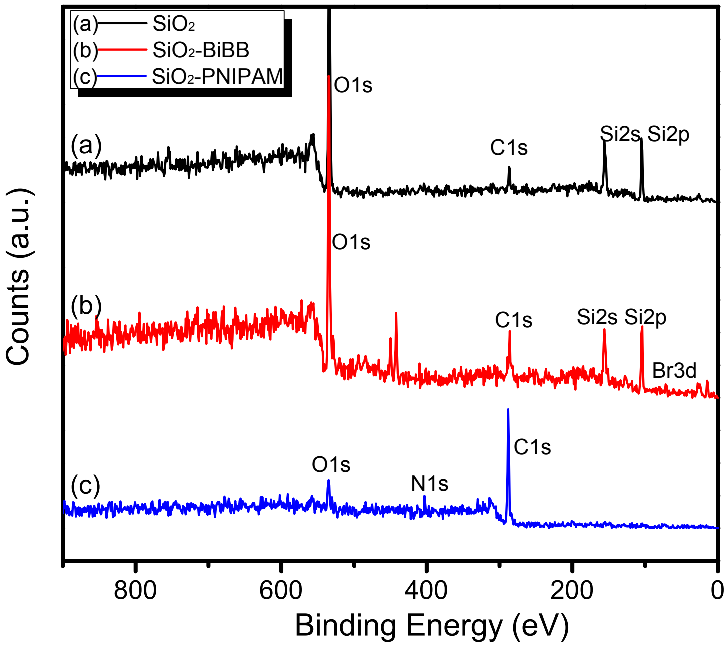

Figure S1. XPS spectra of (**a**) bare silica particles; (**b**) BiBB-functioned silica particles, and (**c**) PNIPAM-grafted silica particles.

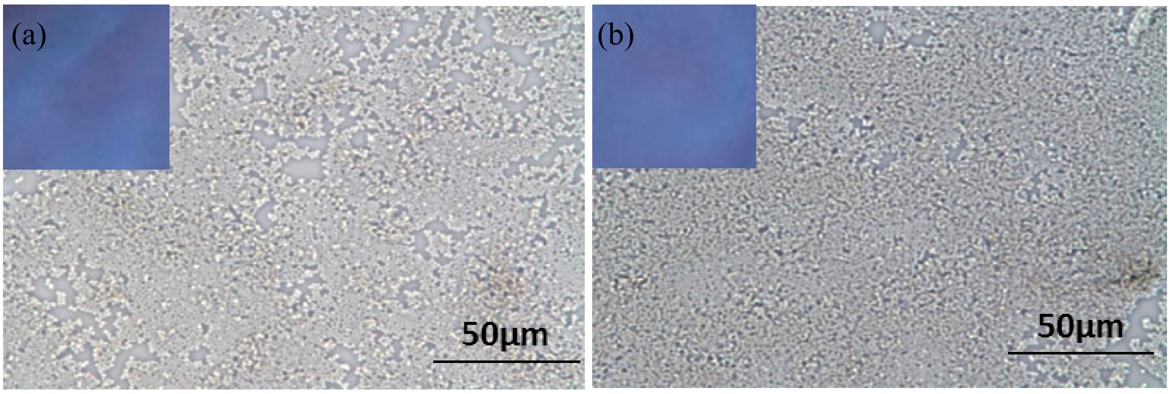

Figure S2. Control experiment: OM images of bare silica microsphere (**a**) below and (**b**) above the LCST of PNIPAM.
